# Supplementary material for: SMAD4 Overexpression in Patients with Sleep Apnoea May Be Associated with Cardiometabolic Comorbidities
Source: J Clin Med. 2020 Jul 25;9(8):2378. doi: 10.3390/jcm9082378 (PMC7464800; doi:10.3390/jcm9082378)
Supplement: Supplementary file 1 [file jcm-09-02378-s001.pdf]

Supplementary Materials

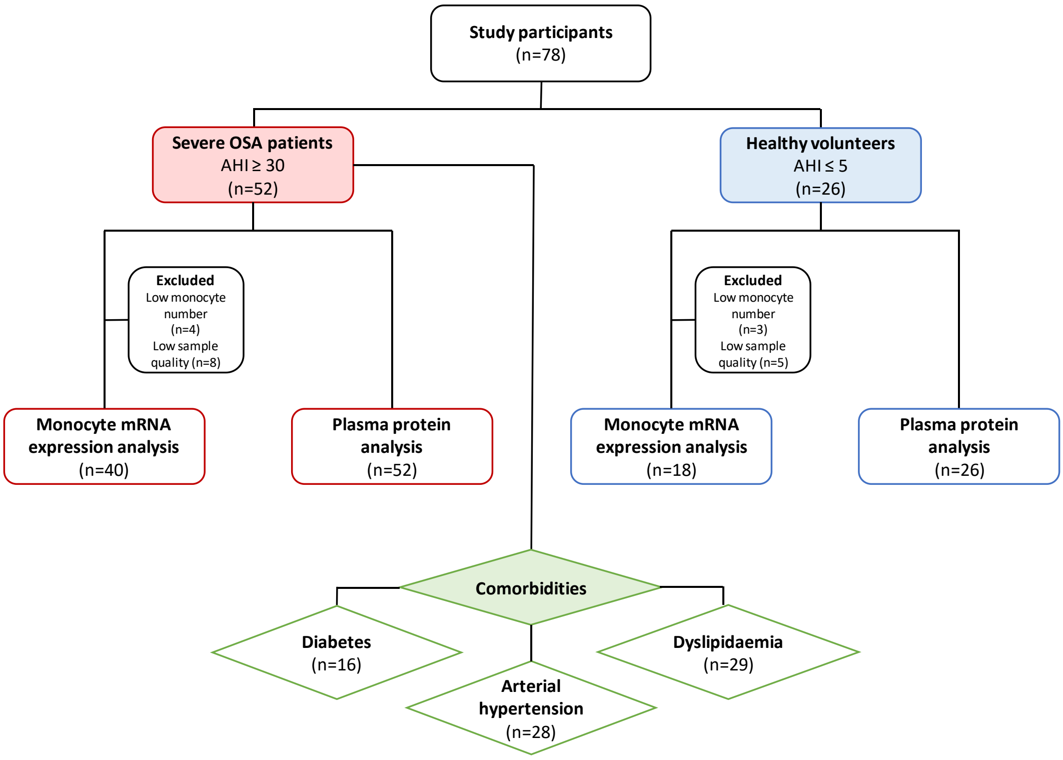

Figure S1. Flow charts of included participants. Subjects were classified in healthy volunteers and OSA patients according to the AHI value. The number of OSA patients that display comorbidities (diabetes, arterial hypertension and dyslipidaemia) and sample distribution are detailed.

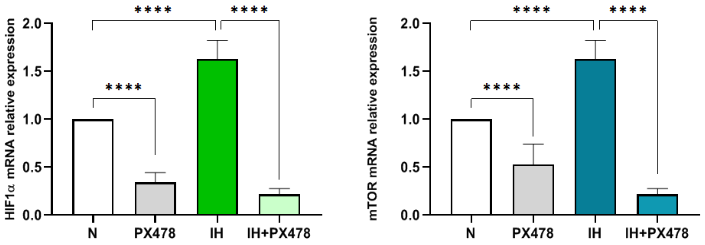

Figure S2. HIF1*α* and mTOR mRNA expression in in-vitro models. HIF1*α* (**left** panel) and mTOR (**right** panel) mRNA expression analysis by qPCR in monocytes from HV subjects (n = 7) treated or not with a specific inhibitor of HIF1*α* (PX-478 15μM) for 16 hours and/or subjected to hypoxia conditions during the same time. Paired control samples were incubated under normoxia conditions and without HIF1*α* inhibitor. Comparisons between groups were performed by one-way ANOVA. Means ± SEM are shown. **** $p < 0.0001$ versus non treated cells are shown.
